# Chloroquine–Primaquine versus Chloroquine Alone to Treat Vivax Malaria in Afghanistan: An Open Randomized Superiority Trial

**DOI:** 10.4269/ajtmh.17-0290

**Published:** 2017-10-30

**Authors:** Ghulam Rahim Awab, Mallika Imwong, Germana Bancone, Atthanee Jeeyapant, Nicholas P. J. Day, Nicholas J. White, Charles J. Woodrow

**Affiliations:** 1 Medical Faculty, Nangarhar University, Jalalabad, Afghanistan;; 2 Ministry of Public Health, Islamic Republic of Afghanistan, Kabul, Afghanistan;; 3 Mahidol-Oxford Tropical Medicine Research Unit (MORU), Faculty of Tropical Medicine, Mahidol University, Bangkok, Thailand;; 4 Department of Molecular Tropical Medicine and Genetics, Faculty of Tropical Medicine, Mahidol University, Bangkok, Thailand;; 5 Shoklo Malaria Research Unit, Mae Sot, Thailand;; 6 Centre for Tropical Medicine and Global Health, Nuffield Department of Medicine, University of Oxford, Oxford, United Kingdom

## Abstract

Afghanistan’s national guidelines recommend primaquine (PQ) for radical treatment of *Plasmodium vivax* malaria, but this is rarely implemented because of concerns over potential hemolysis in patients who have G6PD deficiency. Between August 2009 and February 2014, we conducted an open-label, randomized controlled trial of chloroquine (CQ) alone versus chloroquine plus primaquine (0.25 mg base/kg/day for 14 days) (CQ+PQ) in patients aged 6 months and older with microscopy confirmed *P. vivax* infection. In the CQ+PQ group, G6PD deficiency was excluded by fluorescent spot testing. The primary outcome was *P. vivax* recurrence assessed by survival analysis over one year follow-up. Of 593 patients enrolled, 570 attended at or after 14 days of follow-up. *Plasmodium vivax* recurrences occurred in 37 (13.1%) of 282 patients in the CQ+PQ arm versus 86 (29.9%) of 288 in the CQ arm (Cox proportional hazard ratio [HR] 0.37, 95% confidence interval [CI] 0.25–0.54) (intention-to-treat analysis). Protection against recurrence was greater in the first 6 months of follow-up (HR 0.082; 95% CI 0.029–0.23) than later (HR 0.65, 95% CI 0.41–1.03). Five of seven patients requiring hospital admission were considered possible cases of PQ-related hemolysis, and PQ was stopped in a further six; however, in none of these cases did hemoglobin fall by ≥ 2 g/dL or to below 7 g/dL, and genotyping did not detect any cases of Mediterranean variant G6PD deficiency. PQ 0.25 mg/kg/day for 14 days prevents relapse of *P. vivax* in Afghanistan. Patient visits during the first week may improve adherence. Implementation will require deployment of point-of-care phenotypic tests for G6PD deficiency.

## INTRODUCTION

Malaria remains a significant problem in Afghanistan where cases are caused by *Plasmodium vivax*.^1,2^ Limited resources and security challenges hamper control efforts.^2^ Control of vivax malaria is particularly challenging because the dormant liver stages cause multiple relapses providing a source for new transmission.^3^ Trials of primaquine (PQ), the only widely available radical therapy, in Afghan refugees living in Pakistan documented good efficacy for PQ 0.25 or 0.5 mg/kg/day given over 14 days^4,5^ or 0.75 mg/kg/week for 8 weeks,^5^ compared with a 5-day PQ regimen.^6^ Although adherence to the 14-day treatment course is a concern, efficacy was preserved with an unsupervised regimen accompanied by appropriate instructions.^4^

The national treatment policy for vivax malaria in Afghanistan is chloroquine (CQ) plus 0.25 mg/kg/day PQ for 14 days. Unfortunately, PQ causes hemolysis in individuals with G6PD deficiency, a common red cell enzyme deficiency in malaria endemic regions.^7–9^ G6PD phenotypic tests are generally unavailable in Afghanistan as in many other tropical regions. Hence, administration of PQ according to the national guideline is rarely undertaken.

To assess the effectiveness of unsupervised PQ in preventing relapse of vivax malaria in Afghanistan and thereby support local evidence-based policy development, we compared the relative therapeutic effectiveness of CQ and chloroquine plus primaquine (CQ+PQ) in the treatment of vivax malaria at two provincial sites in Afghanistan.

## MATERIALS AND METHODS

### Study area and participants.

In Afghanistan, malaria is confined to the northern plains, the Jalalabad basin, and valleys fringing the central mountains to the west and south.^10,11^ Malaria is seasonal with cases commencing in May, peaking in July and August, and rare by November.

The study was conducted from August 2009 to February 2014 at two provincial malaria control centers. Jalalabad is a referral center for the whole eastern region. Asadabad is the capital city of Kunar province, a largely mountainous area. Both areas border Pakistan with which there is uncontrolled movement.

### Enrollment.

This prospective, open label, randomized controlled trial was conducted in patients aged 6 months and older with uncomplicated microscopy confirmed symptomatic vivax malaria. Clinical and demographic findings and vital signs were documented, including axillary temperature. Capillary blood was collected for hemoglobin measurement (HemoCue, Ängelholm, Sweden), Giemsa-staining of thick and thin blood films for parasite speciation and counting (40 × number of parasites per 200 white blood cells on the thick film) and G6PD fluorescent spot test (see below). Parasitemia was considered negative after examination of 30 thick film high-power fields.

Inclusion criteria were monoinfection with asexual stages of *P. vivax*, axillary temperature ≥ 37.5°C or oral/rectal temperature ≥ 38°C or history of fever in preceding 24 hours, a negative urine pregnancy test in women at risk of pregnancy, ability to swallow oral medication and comply with study requirements, and written informed consent by the patient or attending parent/guardian. Exclusion criteria were any clinical or laboratory feature of severe malaria,^12^ hemoglobin concentration ≤ 8 g/dL, known G6PD deficiency, significant comorbidity, known hypersensitivity to any study drugs, mixed species *Plasmodium* infection and pregnancy or lactation.

### Randomization.

The two treatment arms were CQ and CQ+PQ. Patients were allocated using a pregenerated randomization list in blocks of 20 held independently of the field teams by a statistician. The individual allocations were kept in sealed envelopes and opened only after enrolment. The microscopists but not the patients and clinical field workers were blinded to treatment arm.

### G6PD phenotypic testing.

Patients randomized to CQ+PQ were tested for G6PD deficiency before receiving antimalarial medication. Samples were tested using the G-6-PD OSMMR 2000 (R&D Diagnostics, Aghia Paraskevi, Greece) NADPH fluorescent spot test kit. When fluorescence was absent (indicating severe deficiency of G6PD) patients were withdrawn and given CQ alone with routine follow-up. Patients with normal fluorescence were treated with CQ+PQ (see below). All patients were advised to return as soon as possible if new symptoms developed (such as dark urine) or their condition worsened significantly. As far as possible, baseline dried blood spots were retained for later testing for the prevalent G6PD Mediterranean variant (563C>T) by polymerase chain reaction (PCR)-restriction fragment length polymorphism.^13^

### Drugs.

Patients received quality-controlled CQ (International Dispensary Association, Amsterdam, The Netherlands) aiming for a target total dose of 25 mg base/kg in divided doses over 3 days, with or without PQ (Bangkok GPO) 0.25 mg base/kg/day for 14 days. According to body weight, individual doses were rounded to the nearest quarter tablet or tablets were crushed, mixed with 5 mL water and the appropriate volume given. CQ and the first three PQ doses were observed at the malaria treatment center and patients remained for 60 minutes after drug administration. Patients vomiting within this time received the same dose again. Patients who vomited the medication twice were withdrawn from the study. Aside from the day 7 PQ dose (observed at the center), the remainder of the PQ doses were taken at home.

### Stopping PQ.

PQ was stopped if there were significant symptoms suggesting hemolysis, hemoglobin concentration fell ≥ 2 g/dL, or absolute hemoglobin was < 7 g/dL at any time. Clinicians were free to stop PQ for clinical reasons outside these criteria. If necessary patients were admitted to hospital (a serious adverse event).

### Follow-up.

Patients were seen daily for the first 3 days, weekly to 28 days and monthly for 12 months.^14,15^ Patients were also requested to attend if they felt unwell at other times during follow-up. At each visit a standard symptom questionnaire was completed. Thick and thin blood slides were examined and hemoglobin measured. Other tests were undertaken as indicated.

Patients with recurrent *P. vivax* infections had capillary blood collected on filter paper for confirmatory PCR analysis and were treated with the same treatment as before (CQ or CQ+PQ); patients in the CQ+PQ arm who had failed to complete the initial PQ course and then experienced recurrent *P. vivax* were treated with CQ only.

Patients were censored from analysis if there was stated withdrawal of consent, severe malaria or persistent vomiting during the acute phase (necessitating parenteral treatment) or development of concomitant disease interfering with classification of treatment outcome. Patients who developed *P. falciparum* infections were treated with artesunate plus sulphadoxine-pyrimethamine^16^ and retained in the study. Patients failing to attend follow-up were visited at home and asked to reattend the center. Remuneration for transportation was provided for follow-up visits. All patients were given one long-lasting insecticide treated bed net.

### Outcomes.

The primary outcome was *P. vivax* recurrence (detected by microscopy) assessed by survival analysis. Secondary outcomes were the total number of recurrences, and the safety and tolerability of the treatments. Patients who completed study treatment entered the per protocol (PP) analysis. Those in the CQ+PQ arm who failed to complete PQ continued to be followed up and were included in the intention-to-treat (ITT) analysis. Patients who did not attend at or after 14 days were excluded from both efficacy analyses.

### Sample size.

It was hypothesized that PQ would reduce the proportion of patients with at least one recurrent attack of *P. vivax* within a year after treatment from 25% to 15%. Using Z-test with continuity correction, one-sided significance level (α) = 0.025 and power = 80%, calculated sample size was 270 per arm; 600 patients were planned to allow for 10% loss to follow up.

### Statistical analysis.

Data were entered onto record forms, transferred electronically to an Open Clinica database and downloaded via Excel into STATA (v10) (Statacorp, College Station, TX). Student’s *t* test, Mann-Whitney U and χ^2^ (or Fisher’s exact) tests were used for comparison of baseline variables, as appropriate. Parasitological failure rates were assessed by recurrence-free survival analysis (Kaplan-Meier). Cox regression was performed to identify possible independent predictors of recurrence, using a stepwise elimination method.

### Species confirmation by PCR.

DNA was extracted from filter-paper blood spots from recurrent cases and nested PCR performed to detect *P. vivax* and *P. falciparum*.^17^

### Ethical approval.

The study was approved by the Ethics Committee of the Faculty of Tropical Medicine, Mahidol University, Thailand, the Oxford Tropical Research Ethics Committee, Oxford University, United Kingdom and the Institutional Review Board of the Afghan Public Health Institute, Ministry of Public Health, Afghanistan. The trial was registered under the identifier NCT01178021.

## RESULTS

### Recruitment.

**Figure 1.**
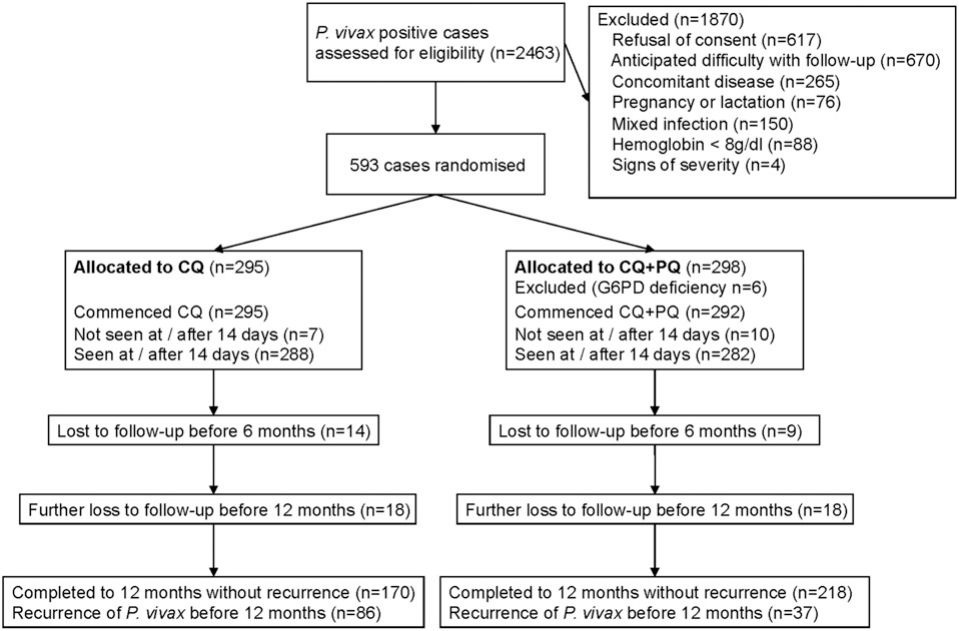
Trial Flow. CQ = chloroquine; CQ+PQ = chloroquine plus primaquine.

Between August 2009 and February 2013, 13,735 patients presented to the study centers with fever. Five hundred and ninety three patients who had monoinfection with *P. vivax* were enrolled in the study and randomized to the CQ (*N* = 295) and CQ+PQ (*N* = 298) arms (Figure 1).

Six patients (five male, one female) randomized to CQ+PQ were judged G6PD deficient on fluorescent spot testing, treated with CQ and withdrawn from the study. Later G6PD molecular testing using retained baseline dried blood spots showed that three males were hemizygous for the Mediterranean deficient variant, while the female was wild-type (genotyping was unsuccessful in one male and one sample was missing).

### Baseline characteristics.

Baseline characteristics were similar between the two treatment groups (Table 1).

**Table 1:** Baseline characteristics of enrolled patients

Characteristic	Treatment arm		
Number of patients starting treatment	CQ (*N* = 295)	CQ+PQ (*N* = 292)	*P*
Sex: male/female (ratio)	200/95 (2.11)	187/105 (1.78)	0.34
Age, median years (range)	15 (2–84)	16 (2–65)	0.16
Weight, median kg (range)	46 (8–100)	52 (8–92)	0.12
Geometric mean parasitemia/µL (95% CI)	2,249 (2,027–2,494)	2,409 (2,185–2,655)	0.33
Number with gametocytes	224 (75.9)	219 (74.2)	0.62
Hemoglobin concentration, mean g/dl (95% CI)	12.0 (11.8–12.2)	12.0 (11.9–12.2)	0.75
Body temperature °C, mean (95% CI)	37.9 (37.8–38.1)	38.2 (37.8–38.5)	0.18
Jaundice	2 (0.7)	1 (0.3)	0.57

CI = confidence interval.

Data for chloroquine (CQ) and chloroquine plus primaquine (CQ+PQ) are number (%) unless otherwise indicated.

### Follow-up.

Of the 570 individuals seen at or after 14 days, 23 (4.0%) were lost to follow-up before 6 months. A total of 59 (10.4%) were lost to follow-up (without experiencing the primary endpoint) before 1 year. Security issues in the Kunar region in 2010 prevented the latter stages of follow-up in a set of 27 consecutive patients recruited at the Kunar site. Two patients had *P. falciparum* monoinfection during follow-up, were treated with artesunate plus sulphadoxine-pyrimethamine, and retained in the study.

### Therapeutic response over 12 months.

**Figure 2.**
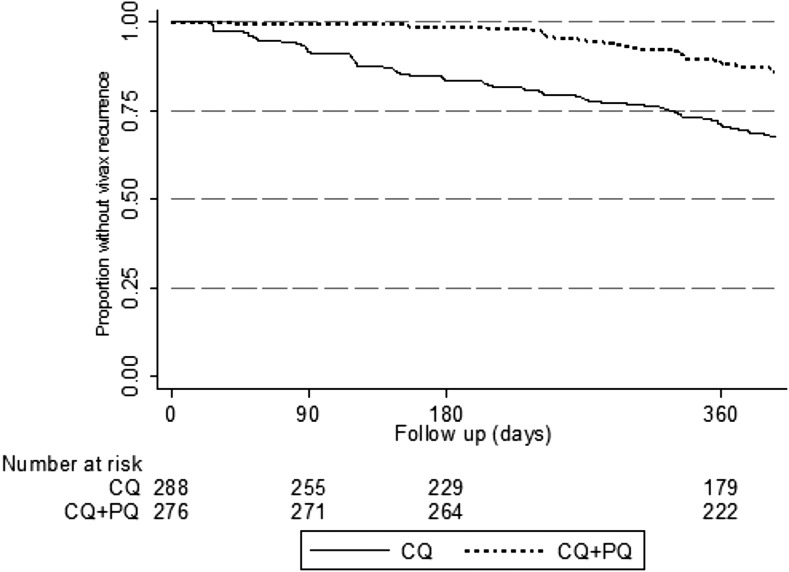
Recurrence-free survival curves: The proportion of subjects free from recurrence of *P. vivax* (based on light microscopy) is displayed according to treatment arm (ITT analysis). CQ = chloroquine; CQ+PQ = chloroquine plus primaquine; ITT analysis = intention-to-treat analysis.

At least one *P. vivax* recurrence occurred in 86 (29.9%) of 288 patients in the CQ arm and 37 (13.1%) of 282 in the CQ+PQ arm (Figure 2). Most (68 and 33, respectively) had a single recurrence, 17 and 4, respectively, had two recurrences and one patient in the CQ arm had three recurrences. Gametocytes were microscopically visible in 70% of recurrences.

The ITT analysis confirmed that recurrences were less common with CQ+PQ (hazard ratio [HR] 0.37, 95% confidence interval [CI] 0.25–0.54). The PP analysis (excluding six patients not completing PQ) gave similar results: CQ+PQ HR 0.35 (0.24–0.52).

Protection from recurrence afforded by PQ was largely confined to the first 6 months of follow-up, with 45 and four patients with recurrence in the CQ and CQ+PQ arms respectively. The Cox proportional estimate for protective effect of PQ in the first 6 months was 0.082 (95% CI 0.029–0.23) by ITT analysis. In months 7–12 protection was no longer significant (HR 0.65, 95% CI 0.41–1.03).

Univariate analysis showed that age, gender, weight, baseline hemoglobin and parasitemia were not significantly associated with recurrence. Baseline gametocytemia on microscopy was associated with recurrence (HR 2.08, 95% CI 1.25–3.48), and remained significant in a multivariable analysis with treatment arm. However, in contrast to the effect of PQ, the association was not significant in the first 6 months (HR 1.64, 95% CI 0.77–3.50) and was only significant for months 6–12 (HR 2.48, 95% CI 1.24–4.99).

### Parasite clearance and early recurrence.

At day 1 after treatment, 84.3% of patients in the CQ arm and 90.3% in the CQ+PQ arm had cleared parasites on the peripheral blood film; by day 2, this proportion was 98.6% in both arms. Eight patients (1.4%: 7 in the CQ arm) had recurrent *P. vivax* before day 42 with six in the CQ arm occurring at day 28 (or 1 day before). The median parasitemia at recurrence in these six cases was 1,980/μL (hence similar to the overall study).

### Molecular speciation of recurrent isolates.

Dried blood spots from reported *P. vivax* recurrences were speciated by PCR. Of 88 available samples, 79 were found to be *P. vivax*, four *Plasmodium falciparum* and two mixed infections (three cases had no amplification product). Of the *P. falciparum* monoinfections (misidentified as *P. vivax*), three were in the CQ arm and one in the CQ+PQ arm; one of these CQ cases went on to have a true *P. vivax* recurrence (confirmed by PCR). Inaccurately speciated cases are hence unlikely to have significantly impacted the main findings.

### Hemoglobin changes.

**Figure 3.**
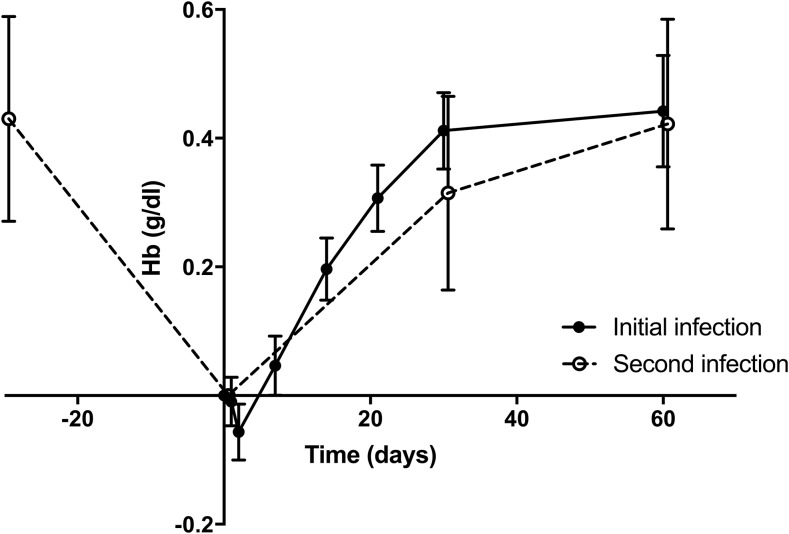
Hemoglobin changes associated with *Plasmodium vivax* infection. Data are stratified according to whether the infection was at study recruitment or recurrence.

Hemoglobin concentrations after enrollment recovered over the course of 2 months, with a mean (95% CI) increase compared with baseline of 0.44 g/dL (0.36–0.53) (Figure 3). Recurrent *P. vivax* was associated with a fall and rise in hemoglobin of very similar magnitude (fall compared with previous infection-free visit = 0.43 g/dL, 0.27–0.59; rise 2 months after recurrence = 0.42 g/dL, 0.26–0.58) (Figure 3).

### Adverse events.

Both treatments were generally well tolerated. Repeated vomiting of the study medication was not reported. Seven patients deteriorated, requiring hospital admission, during the treatment phase and were withdrawn from the study (Table 2); five were considered possibly related to PQ in the CQ+PQ arm. However, in none of these cases did hemoglobin fall by ≥ 2 g/dL, or to below 7 g/dL and G6PD genotyping, possible in five cases, indicated only wild-type alleles at the Mediterranean G6PD deficiency locus (Table 2).

**Table 2:** Serious adverse events and cases in which primaquine was stopped

Arm	Gender	Syndrome	Relation to study drug	Hb d0	Hb d1	Hb d2	Hb d7	G6PD genotype*	Action
Serious adverse events									
CQ	F	Vomiting	Unlikely	9.3	9.2	–	–	WT	Hospitalized d1
CQ+PQ	F	Jaundice	Possible	13	13	11	–	WT	Hospitalized d2
CQ+PQ	M	Dyspnoea	Possible	14.2	–	13.5	13.2	–	Hospitalized d7
CQ+PQ	M	Jaundice, dark oliguria	Possible	9.8	–	9.2	8.5	–	Hospitalized d2
CQ+PQ	F	Anemia, tachycardia	Possible	10.4	–	10.5	9.8	WT	Hospitalized d7
CQ+PQ	F	Hypotension, dark urine, jaundice	Possible	13.0	12.0	12.0	12.0	WT	Hospitalized d7
CQ+PQ	F	Vomiting, food poisoning	Unlikely	10.2	–	–	–	WT	Hospitalized d1
Other cases where PQ was stopped or jaundice described									
CQ	F	Jaundice, dizziness	Possible	9.0	8.6	8.5	8.6	WT	No action taken
CQ+PQ	M	Face swelling, dyspnea, dark urine	Possible	13.2	13.2	12.5	13.4	WT	PQ stopped d1
CQ+PQ	M	Dark urine	Possible	14.5	–	–	12.8	–	PQ stopped d7
CQ+PQ	M	Jaundice, dark urine	Possible	9.2	–	9.4	10.0	WT	PQ stopped d2
CQ+PQ	M	Jaundice, dark urine, dyspnoea	Possible	10.4	–	9.8	10.2	–	PQ stopped d2
CQ+PQ	F	Jaundice, dark oliguria, dyspnoea	Possible	9.3	–	9.0	8.8	WT	PQ stopped d7
CQ+PQ	M	Jaundice, dark urine, dyspnoea	Possible	8.8	–	8.7	7.5	WT	PQ stopped d7

CQ = chloroquine; PQ = primaquine.

* G6PD genotype (hemizygous or homozygous) at the Mediterranean locus (563C>T mutation).

There were seven other cases where signs or symptoms considered compatible with hemolysis occurred but the patient was not hospitalized or withdrawn. Six of these were in the CQ+PQ arm, and PQ administration was stopped (Table 2). Some patients were anemic before treatment. G6PD genotyping (possible in five cases) indicated only wild-type alleles at the Mediterranean locus.

## DISCUSSION

The trial’s primary aim was to measure the efficacy of unsupervised PQ (14 days at 0.25 mg/kg/day) as radical treatment of *P. vivax* in Afghanistan. Measuring the efficacy of radical treatment involves consideration of several methodological issues. Molecular tests cannot distinguish relapse from reinfection or recrudescence (a problem if there is resistance to the initial schizonticidal treatment), requiring a control arm of CQ, efficacious only against the blood-stage infection. The follow-up period needs to take into account the natural history of relapses in the given location.^18^ In Southeast Asia and Oceania, where relapses are frequent and early, differences in relapse rate can be discerned with a relatively short period of follow-up. In South Asia, relapse rates are lower, and in some areas, long latency temperate strains are prevalent,^19^ so a longer period of observation with high rates of follow-up is required to obtain sufficient power.

Given its high rate of follow-up, our study provided a clear view of the natural history of vivax relapse in Afghanistan. The survival curves in the two study arms appeared to diverge from the outset, with seven of eight early recurrences (before day 42) in the CQ only arm. PQ has significant blood-stage activity,^20^ but still the lack of early infections in the CQ+PQ arm suggests that these were relapses rather than recrudescences, indicating ongoing efficacy of CQ against blood stages.^21^ For the first 6 months, the recurrence-free survival curve for CQ indicated a constant recurrence frequency of about 2.5% per month; again, these are likely to have been relapses because recurrences remained rare in the CQ+PQ arm. After 6 months, the two survival curves became more similar, suggesting that reinfections predominated, although there was a possible excess of recurrences in the CQ group after 8 months that may represent late relapses of long latency infections as seen previously^5,19^ (although numbers are too small for clear definition). The overall recurrence rates in the two arms are broadly similar to those previously found in Afghan refugees in Pakistan.^2^

The overall efficacy of 0.25 mg/kg/day PQ for 14 days over a year was approximately 65%. The efficacy during the first 6 months, in which relapses are likely to have predominated, was around 90%. This study hence provides clear evidence that the current PQ regimen is efficacious in radical cure. The total dose of 3.5 mg/kg prescribed is categorized as “low dose” according to the stratification proposed by John et al.^18^ and has been shown to provide useful antirelapse activity (of varying magnitude) in other randomized controlled studies undertaken in Pakistan,^4^ India,^22,23^ Thailand,^24,25^ and Ethiopia^26^ as well as the multi-center DETECTIVE study^27^; these studies have also been summarized in relevant meta-analyses.^18,28,29^

Vivax recurrence was associated with a fall of approximately 0.5 g/dL hemoglobin. Although unlikely to be clinically detrimental in isolation, this could carry a cumulative effect with repeated recurrences (particularly if diagnosed late and/or associated with other forms of anemia).^30^

CQ has been used as first-line schizonticidal treatment of vivax malaria in Afghanistan for many decades. The data obtained in this study suggest that it remains an efficacious schizonticide in this region. Six cases in the CQ arm experienced relapse at 28 days (or just before). Without drug levels at the time of recurrence it is not possible to exclude the possibility that these cases represent CQ-resistant parasites, but the overall proportion of such cases (well under 5% of the total) is indicative of a sensitive population^31^ and consistent with previous studies.^5,6,21^ In addition, microscopic parasite clearance rate was fast in both CQ and CQ+PQ arms, with more than 98% of cases clear of parasites by day two, another marker of CQ sensitivity.^31^

The study had certain weaknesses which might impact on its generalizability. Most PQ doses were unsupervised, but PQ was observed on days zero, one, two, and seven, with further reinforcement of adherence by ongoing follow-up, so it was partly supervised, potentially overestimating effectiveness. However, previous work suggests good levels of adherence in this community.^4^

The work was undertaken in a resource-poor setting with challenging logistic and security issues; at one stage, the Kunar site closed because of civil unrest. The malaria treatment centers lack facilities for detailed assessment of possible hemolysis, leading to the possibility of subjective decision making by staff. Linked to this, the study was nonblinded, with clinical staff aware of treatment allocation. None of the hospitalized patients judged to have a syndrome compatible with PQ-induced hemolysis, or in whom PQ was stopped, met laboratory criteria for hemolysis, suggesting that these were not hemolytic episodes; furthermore, retrospective G6PD genotyping (limited to the Mediterranean locus) indicated only wild-type alleles. Notably, some of these patients were anemic at baseline, suggesting that study doctors may have been influenced by disease rather than treatment effects. Use of placebo would have offset this problem.^4,32^

For pragmatic reasons, detection and speciation of recurrent malaria, clearly central to assessment of the primary endpoint, were undertaken by microscopy, thereby focusing on the efficacy of treatment in terms of symptomatic cases. Detection of recurrence via the more sensitive technique of PCR (on dried blood spots) would have allowed assessment of the impact of PQ on all recurrences, including submicroscopic infections which can increase the risk of anemia^30^ and also contribute to the infectious reservoir in low transmission settings.^33,34^ Molecular validation by PCR of dried blood spots indicated that a small proportion (around 5%) of recurrent infections thought to be *P. vivax* were actually *P. falciparum*, an unsurprising result given previously identified challenges in accuracy of malaria diagnosis in Afghanistan.^35^ The relatively low number of possibly misclassified cases appears unlikely to have compromised the study’s overall conclusions.

## CONCLUSION

PQ administered in a largely unsupervised fashion at 0.25 mg/kg/day for 14 days provides efficacious radical cure in *P. vivax* patients in Afghanistan. Because of ongoing safety concerns, methods for exclusion of G6PD deficiency that can be applied a range of field settings^36–38^ are needed if PQ is to be widely deployed.

## References

[1] Kolaczinski J Graham K Fahim A Brooker S Rowland M, 2005 Malaria control in Afghanistan: progress and challenges. Lancet 365: 1506–1512. 15850637 10.1016/S0140-6736(05)66423-9

[2] Leslie T Nahzat S Sediqi W, 2016 Epidemiology and control of *Plasmodium vivax* in Afghanistan. Am J Trop Med Hyg 95: 72–77. 27708189 10.4269/ajtmh.16-0172 PMC5201225

[3] Garnham PC, 1988 Swellengrebel lecture. Hypnozoites and ‘relapses’ in *Plasmodium vivax* and in vivax-like malaria. Trop Geogr Med 40: 187–195. 3055568

[4] Leslie T Rab MA Ahmadzai H Durrani N Fayaz M Kolaczinski J Rowland M, 2004 Compliance with 14-day primaquine therapy for radical cure of vivax malaria–a randomized placebo-controlled trial comparing unsupervised with supervised treatment. Trans R Soc Trop Med Hyg 98: 168–173. 15024927 10.1016/s0035-9203(03)00041-5

[5] Leslie T Mayan I Mohammed N Erasmus P Kolaczinski J Whitty CJ Rowland M, 2008 A randomised trial of an eight-week, once weekly primaquine regimen to prevent relapse of *Plasmodium vivax* in Northwest Frontier Province, Pakistan. PLoS One 3: e2861. 18682739 10.1371/journal.pone.0002861 PMC2481394

[6] Rowland M Durrani N, 1999 Randomized controlled trials of 5- and 14-days primaquine therapy against relapses of vivax malaria in an Afghan refugee settlement in Pakistan. Trans R Soc Trop Med Hyg 93: 641–643. 10717755 10.1016/s0035-9203(99)90081-0

[7] Bouma MJ Goris M Akhtar T Khan N Khan N Kita E, 1995 Prevalence and clinical presentation of glucose-6-phosphate dehydrogenase deficiency in Pakistani Pathan and Afghan refugee communities in Pakistan; implications for the use of primaquine in regional malaria control programmes. Trans R Soc Trop Med Hyg 89: 62–64. 7747310 10.1016/0035-9203(95)90661-4

[8] Leslie T Briceño M Mayan I Mohammed N Klinkenberg E Sibley CH Whitty CJ Rowland M, 2010 The impact of phenotypic and genotypic G6PD deficiency on risk of *Plasmodium vivax* infection: a case-control study amongst Afghan refugees in Pakistan. PLoS Med 7: e1000283. 20520804 10.1371/journal.pmed.1000283 PMC2876136

[9] Jamornthanyawat N Awab GR Tanomsing N Pukrittayakamee S Yamin F Dondorp AM Day NP White NJ Woodrow CJ Imwong M, 2014 A population survey of the glucose-6-phosphate dehydrogenase (G6PD) 563C>T (Mediterranean) mutation in Afghanistan. PLoS One 9: e88605. 24586352 10.1371/journal.pone.0088605 PMC3931629

[10] Faulde MK Hoffmann R Fazilat KM Hoerauf A, 2007 Malaria reemergence in northern Afghanistan. Emerg Infect Dis 13: 1402–1404. 18252122 10.3201/eid1309.061325 PMC2857272

[11] Dhir SL Rahim A, 1957 Malaria and its control in Afghanistan (1950–1954). Indian J Malariol 11: 73–126. 13438535

[12] WHO, 2000 Severe and complicated malaria. Trans R Soc Trop Med Hyg 94 (*Suppl 1*): 1–90.10748883

[13] Dell’edera D 2012 Study of a family in the province of Matera presenting with glucose-6-phosphate dehydrogenase deficiency and Gilbert’s syndrome. Mol Med Rep 5: 1521–1525. 22407023 10.3892/mmr.2012.830

[14] Stepniewska K White NJ, 2006 Some considerations in the design and interpretation of antimalarial drug trials in uncomplicated falciparum malaria. Malar J 5: 127. 17187673 10.1186/1475-2875-5-127 PMC1769507

[15] Pukrittayakamee S Chantra A Simpson JA Vanijanonta S Clemens R Looareesuwan S White NJ, 2000 Therapeutic responses to different antimalarial drugs in vivax malaria. Antimicrob Agents Chemother 44: 1680–1685. 10817728 10.1128/aac.44.6.1680-1685.2000 PMC89932

[16] Awab GR Imwong M Pukrittayakamee S Alim F Hanpithakpong W Tarning J Dondorp AM Day NP White NJ Woodrow CJ, 2016 Clinical trials of artesunate plus sulfadoxine-pyrimethamine for *Plasmodium falciparum* malaria in Afghanistan: maintained efficacy a decade after introduction. Malar J 15: 121. 26917051 10.1186/s12936-016-1167-z PMC4766631

[17] Snounou G, 1996 Detection and identification of the four malaria parasite species infecting humans by PCR amplification. Methods Mol Biol 50: 263–291. 8751365 10.1385/0-89603-323-6:263

[18] John GK Douglas NM von Seidlein L Nosten F Baird JK White NJ Price RN, 2012 Primaquine radical cure of *Plasmodium vivax*: a critical review of the literature. Malar J 11: 280. 22900786 10.1186/1475-2875-11-280 PMC3489597

[19] Kim JR Nandy A Maji AK Addy M Dondorp AM Day NP Pukrittayakamee S White NJ Imwong M, 2012 Genotyping of *Plasmodium vivax* reveals both short and long latency relapse patterns in Kolkata. PLoS One 7: e39645. 22808048 10.1371/journal.pone.0039645 PMC3396609

[20] Pukrittayakamee S Vanijanonta S Chantra A Clemens R White NJ, 1994 Blood stage antimalarial efficacy of primaquine in *Plasmodium vivax* malaria. J Infect Dis 169: 932–935. 8133114 10.1093/infdis/169.4.932

[21] Awab GR Pukrittayakamee S Imwong M Dondorp AM Woodrow CJ Lee SJ Day NP Singhasivanon P White NJ Kaker F, 2010 Dihydroartemisinin-piperaquine versus chloroquine to treat vivax malaria in Afghanistan: an open randomized, non-inferiority, trial. Malar J 9: 105. 20409302 10.1186/1475-2875-9-105 PMC2864284

[22] Gogtay NJ Desai S Kamtekar KD Kadam VS Dalvi SS Kshirsagar NA, 1999 Efficacies of 5- and 14-day primaquine regimens in the prevention of relapses in *Plasmodium vivax* infections. Ann Trop Med Parasitol 93: 809–812. 10715673 10.1080/00034989957790

[23] Rajgor DD Gogtay NJ Kadam VS Kamtekar KD Dalvi SS Chogle AR Aigal U Bichile LS Kain KC Kshirsagar NA, 2003 Efficacy of a 14-day primaquine regimen in preventing relapses in patients with *Plasmodium vivax* malaria in Mumbai, India. Trans R Soc Trop Med Hyg 97: 438–440. 15259476 10.1016/s0035-9203(03)90082-4

[24] Luxemburger C van Vugt M Jonathan S McGready R Looareesuwan S White NJ Nosten F, 1999 Treatment of vivax malaria on the western border of Thailand. Trans R Soc Trop Med Hyg 93: 433–438. 10674098 10.1016/s0035-9203(99)90149-9

[25] Walsh DS Wilairatana P Tang DB Heppner DG Brewer TG Krudsood S Silachamroon U Phumratanaprapin W Siriyanonda D Looareesuwan S, 2004 Randomized trial of 3-dose regimens of tafenoquine (WR238605) versus low-dose primaquine for preventing *Plasmodium vivax* malaria relapse. Clin Infect Dis 39: 1095–1103. 15486831 10.1086/424508

[26] Yeshiwondim AK Tekle AH Dengela DO Yohannes AM Teklehaimanot A, 2010 Therapeutic efficacy of chloroquine and chloroquine plus primaquine for the treatment of *Plasmodium vivax* in Ethiopia. Acta Trop 113: 105–113. 19835832 10.1016/j.actatropica.2009.10.001

[27] Llanos-Cuentas A 2014 Tafenoquine plus chloroquine for the treatment and relapse prevention of *Plasmodium vivax* malaria (DETECTIVE): a multicentre, double-blind, randomised, phase 2b dose-selection study. Lancet 383: 1049–1058. 24360369 10.1016/S0140-6736(13)62568-4

[28] Galappaththy GN Omari AA Tharyan P, 2007 Primaquine for preventing relapses in people with *Plasmodium vivax* malaria. Cochrane Database Syst Rev CD004389. 17253504 10.1002/14651858.CD004389.pub2

[29] Galappaththy GN Tharyan P Kirubakaran R, 2013 Primaquine for preventing relapse in people with *Plasmodium vivax* malaria treated with chloroquine. Cochrane Database Syst Rev 10: CD004389. 10.1002/14651858.CD004389.pub3 PMC6532739 24163057

[30] Pava Z 2016 Submicroscopic and asymptomatic *Plasmodium* parasitaemia associated with significant risk of anaemia in Papua, Indonesia. PLoS One 11: e0165340. 27788243 10.1371/journal.pone.0165340 PMC5082812

[31] Price RN von Seidlein L Valecha N Nosten F Baird JK White NJ, 2014 Global extent of chloroquine-resistant *Plasmodium vivax*: a systematic review and meta-analysis. Lancet Infect Dis 14: 982–991. 25213732 10.1016/S1473-3099(14)70855-2 PMC4178238

[32] Improv Study Group, 2015 Improving the radical cure of vivax malaria (IMPROV): a study protocol for a multicentre randomised, placebo-controlled comparison of short and long course primaquine regimens. BMC Infect Dis 15: 558. 26643116 10.1186/s12879-015-1276-2 PMC4672497

[33] Cheng Q Cunningham J Gatton ML, 2015 Systematic review of sub-microscopic *P. vivax* infections: prevalence and determining factors. PLoS Negl Trop Dis 9: e3413. 25569135 10.1371/journal.pntd.0003413 PMC4288718

[34] Tadesse FG 2017 The shape of the iceberg: quantification of submicroscopic *Plasmodium falciparum* and *Plasmodium vivax* parasitaemia and gametocytaemia in five low endemic settings in Ethiopia. Malar J 16: 99. 28253867 10.1186/s12936-017-1749-4 PMC5335517

[35] Leslie T Mikhail A Mayan I Anwar M Bakhtash S Nader M Chandler C Whitty CJ Rowland M, 2012 Overdiagnosis and mistreatment of malaria among febrile patients at primary healthcare level in Afghanistan: observational study. BMJ 345: e4389. 22833603 10.1136/bmj.e4389 PMC3404186

[36] Kim S 2011 Performance of the CareStart™ G6PD deficiency screening test, a point-of-care diagnostic for primaquine therapy screening. PLoS One 6: e28357. 22164279 10.1371/journal.pone.0028357 PMC3229584

[37] Bancone G Chu CS Chowwiwat N Somsakchaicharoen R Wilaisrisak P Charunwatthana P Bansil P McGray S Domingo GJ Nosten FH, 2015 Suitability of capillary blood for quantitative assessment of G6PD activity and performances of G6PD point-of-care tests. Am J Trop Med Hyg 92: 818–824. 25646252 10.4269/ajtmh.14-0696 PMC4385780

[38] Satyagraha AW 2016 Assessment of point-of-care diagnostics for G6PD deficiency in malaria endemic rural eastern Indonesia. PLoS Negl Trop Dis 10: e0004457. 26894297 10.1371/journal.pntd.0004457 PMC4760930

